# High Performance on a Pragmatic Task May Not Be the Result of Successful Reasoning: On the Importance of Eliciting Participants’ Reasoning Strategies

**DOI:** 10.1162/opmi_a_00077

**Published:** 2023-06-01

**Authors:** Alexandra Mayn, Vera Demberg

**Affiliations:** Department of Language of Science and Technology, Saarland University; Department of Computer Science, Saarland University

**Keywords:** experimental stimuli, reasoning, pragmatic inferences

## Abstract

Formal probabilistic models, such as the Rational Speech Act model, are widely used for formalizing the reasoning involved in various pragmatic phenomena, and when a model achieves good fit to experimental data, that is interpreted as evidence that the model successfully captures some of the underlying processes. Yet how can we be sure that participants’ performance on the task is the result of successful reasoning and not of some feature of experimental setup? In this study, we carefully manipulate the properties of the stimuli that have been used in several pragmatics studies and elicit participants’ reasoning strategies. We show that certain biases in experimental design inflate participants’ performance on the task. We then repeat the experiment with a new version of stimuli which is less susceptible to the identified biases, obtaining a somewhat smaller effect size and more reliable estimates of individual-level performance.

## INTRODUCTION

Formal probabilistic models, such as the Rational Speech Act model (RSA, Frank & Goodman, 2012), are widely used to formalize the reasoning involved in various pragmatic phenomena, such as scalar implicatures (Goodman & Stuhlmüller, 2013), hyperbole (Kao et al., 2014), and irony (Kao & Goodman, 2015). RSA assumes that the speaker and the listener are cooperative and reason recursively about each other to arrive at a shared interpretation. These models are then evaluated by being fitted to experimental data, and if the they achieve a close fit, that is interpreted as evidence that the model successfully captures some of the underlying processes.

Yet how can we be sure that participants’ high performance on the task is indeed the result of successful reasoning, and not some other factor related to the experimental setup? Sikos et al. (2021) revisited the influential RSA study by Frank and Goodman (2012) and found only modest evidence of people engaging in the assumed pragmatic reasoning. They showed that a simple literal listener model, which is governed by the salience prior over objects, provided an equally good fit to the data as the full pragmatic listener model. The authors therefore argued that the good fit of the RSA model to empirical data was largely due to a combination of non-pragmatic factors. Neglecting to carefully investigate whether the experimental design contains biases hence may lead to making unwarranted conclusions about the phenomena we study.

Franke and Degen (2016) ask their participants to identify the referent of an ambiguous message and show that there is quite a lot of individual variability in performance, and that the data is better captured by a model which assumes that each participant has their own reasoning type, corresponding to predictions of three probabilistic models of different degrees of complexity, than by a population-level model that assumes that all participants have the same reasoning. Therefore, better performance on the task is interpreted as evidence of participants successfully employing higher complexity of reasoning.

In this study, we conduct a series of experiments based on the task used by Franke and Degen (2016) (originally introduced in Degen & Franke, 2012) where we carefully manipulate the properties of the stimuli and also elicit participants’ reasoning strategies. We argue that certain biases are present in the task which allow participants to arrive at the correct answer without engaging in the assumed pragmatic reasoning. We then design a version of stimuli aimed at mitigating the identified biases and repeat the experiment, obtaining a somewhat smaller effect size, and, importantly, more reliable individual-level results. We argue that probing an experimental design for biases is crucial for ensuring that we can draw meaningful conclusions, and strategy elicitation is a simple and efficient way of doing so.

All data and analysis scripts for the results reported in this paper are available at https://github.com/sashamayn/refgame_stimuli_methods.

## BACKGROUND

### Reference Game

The task which our experiments build on is the reference game, which is the Experiment 1 in Degen and Franke (2012). Participants’ task is to identify the referent of a message.

On each trial, participants are presented with three objects, each of which is a creature wearing an accessory. There are three possible creatures (green monster, purple monster, and robot) and three possible accessories (red hat, blue hat, and scarf). We can think of the objects on the screen, therefore, as varying across two feature dimensions—the creature feature and the accessory feature. Participants also see a message that they are told was sent by the previous participant. The message is always either a creature (without an accessory) or an accessory. Importantly, participants are told that not all creatures and accessories are available as messages: there are no messages *scarf* or *robot*, hence these are so-called *inexpressible* features. Participants’ task is then to pick the creature they believe the previous participant was referring to.

The task consists of 66 experimental trials, of which 24 are critical and 42 are fillers. Each trial display consists of the target (correct answer), competitor and distractor, presented in random order.

**Figure 1.**
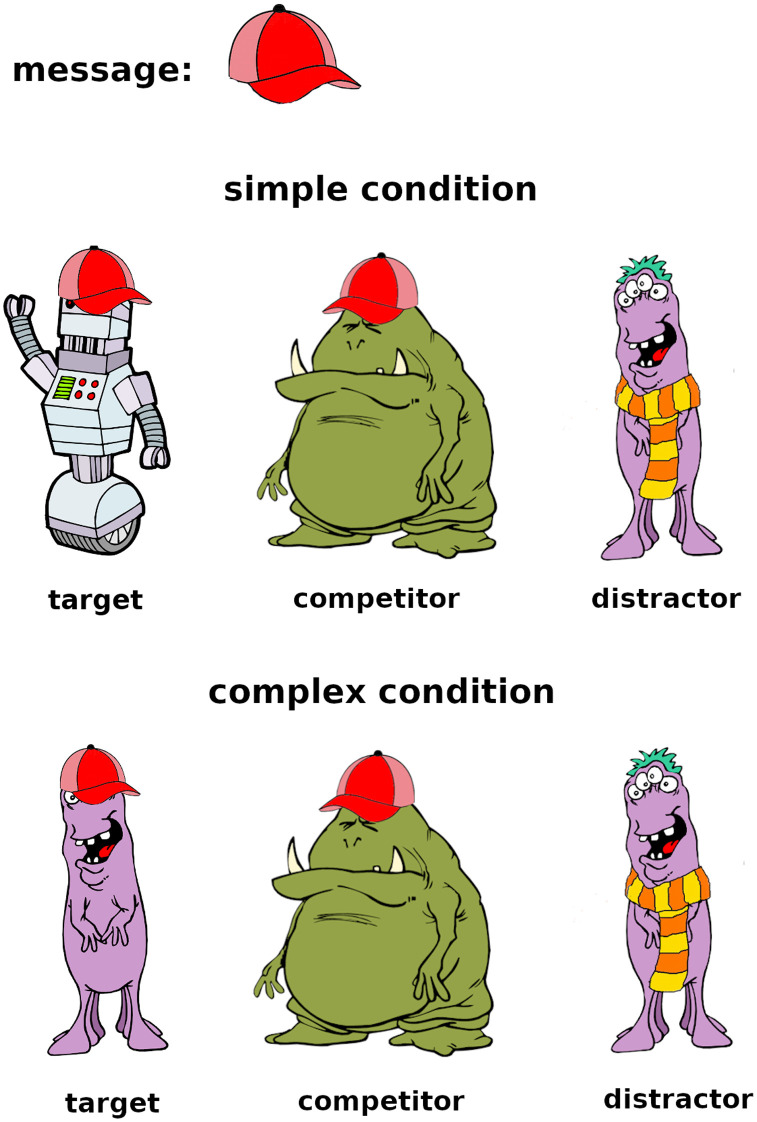
Example of a simple and a complex critical trial.

On the critical trials, the message is ambiguous. Half of the critical trials are *simple implicature trials*, where only one reasoning step is required to solve them. An example of a simple implicature trial is presented in the top panel of Figure 1: the message (red hat) may at first glance be referring to either the robot (target) or to the green monster (competitor). To draw the implicature, one can reason that if the speaker had meant to refer to the competitor (green monster), they could have used the unambiguous message *green monster*, whereas there is no way to refer to the target (robot) unambiguously since *robot* is not an available message, hence the red hat must be referring to the robot.

Complex trials require two reasoning steps because the target shares one feature with the competitor and the other feature with the distractor. An example of a complex implicature trial is presented in the bottom panel of Figure 1. Here, the message (red hat) may at first glance also be referring to either the target (purple monster with a red hat) or the competitor (green monster with a red hat). One would then need to reason that if the speaker had wanted to refer to the competitor (green monster), they could have used the unambiguous *green monster*. In contrast, there is no unambiguous way of referring to the target since its other feature (purple monster) is shared with the distractor, hence the red hat must be referring to the purple monster with the red hat.

Of the 42 fillers, 33 are completely unambiguous and 9 completely ambiguous. On completely unambiguous trials, only the target has the feature expressed by the message. For example, the message is a purple monster and there’s only one purple monster on the screen. Those trials are used as an attention check. On completely ambiguous trials, the target and the competitor are identical. For example, the message is a purple monster and there are two identical purple monsters wearing a blue hat and a robot with a red hat on the screen. Since there is no way of knowing which of the two identical creatures is the target, performance on the ambiguous trials constitutes a random baseline.

### Models of Reasoning Complexity in the Reference Game

Franke and Degen (2016) show that formal probabilistic RSA models make distinct predictions with regards to simple and complex implicatures in the reference game. These models are defined in terms of a speaker *L*_*N*_ and a listener *S*_*N*−*1*_ who recursively reason about each other to arrive at an interpretation. The listener’s task is to identify the referent object *o* given the message *m* uttered by the speaker.

The most simple listener model, the literal listener (*L*_0_), assigns equal probability to every object for which the message is literally true: *L*_0_(*o*|*m*) ∝ *exp*(*λ* ⋅ 𝒰(*o*|{*o*′|*m* is true of *o*′})), where 𝒰 is a uniform distribution and is a hyperparameter which governs how strictly an agent adheres to their utility function as opposed to picking a referent at random. Thus, *L*_0_ will have chance-level performance on both types of critical trials. For example, in the top panel of Figure 1, *L*_0_ will assign equal probability to the target and the competitor since they are both wearing a red hat.

A slightly more sophisticated *L*_1_ listener, who reasons about a literal speaker *S*_0_, will be able to successfully solve the simple but not the complex implicature condition. Let’s show why that is the case. To describe an object *o*, the literal speaker *S*_0_ will pick any message *m* which is literally true of *o* with equal probability: *S*_0_(*m*|*o*) ∝ *exp*(*λ* ⋅ 𝒰(*m*|{*m*′|*m*′ is true of *o*})). Thus, for the top panel of Figure 1, the literal speaker will always use the message “red hat” to refer to the target, and will use the messages “red hat” and “green monster” with equal probability to refer to the competitor. The *L*_1_ listener will then reason that the target is more likely than the competitor, since for the competitor, the *S*_0_’s probability is split between two messages. *L*_1_ is not going to be powerful enough to solve the complex implicatures, however, because for both target and competitor, *S*_0_’s probability of using the *red hat* message to refer to them is $\frac{1}{2}$, as for each of them there is another available message (purple monster and green monster respectively).

Finally, the pragmatic *L*_2_ listener, who reasons about a pragmatic speaker *S*_1_, who, in turn, reasons about the literal listener *L*_0_, is powerful enough to also solve the complex implicature type. That is because, while *S*_0_ is equally likely to use either feature to refer to the target and the competitor, in the *S*_1_, that symmetry is broken. Let’s look at the example in the bottom panel of Figure 1. *S*_1_ knows that *L*_0_ will assign equal probabilityto all referents compatible with the message: if the speaker sends the message *green monster*, the listener will umabiguously identify the competitor, whereas if they send the message *red hat*, the listener will randomly choose one of twofitting referents. Therefore, *S*_1_ will avoid using the red hat to refer to the competitor. The pragmatic listener *L*_2_ knows this, so they will correctly identify the target upon receipt of the message *red hat*.

Franke and Degen (2016) fit a hierarchical Bayesian model to their experimental data from the reference game, whereby one of the three listener types (*L*_0_, *L*_1_ or *L*_2_) was assigned to each individual participant, and showed that this individual-level model provided a better fit to the data than a model that assumed that all participants had the same reasoning type. Mayn and Demberg (2022) found that participants’ performance on the reference game is modulated by their abstract reasoning ability, as measured by the Raven’s Progressive Matrices and the Cognitive Reflection Test.

### Annotation of Reasoning Strategies

Since we were interested in how the participants solve the reference game, in addition to collecting participants responses to the experimental stimuli, in each of our experiments, we also elicited their reasoning strategies. After completing the main experiment, participants saw one simple and one complex item again, randomly selected and presented in randomized order. When they clicked on one of the creatures, a red box appeared around it, along with the question “Why did you make that choice?” and a textbox. This was done as a probe into participants’ reasoning.

All responses were annotated by two annotators, one of whom was blind to the purpose of the experiment. All disagreements were resolved jointly: the two annotators met and each made a case for their choice of tag. If one of the annotators successfully convinced the other, the tag was changed accordingly; otherwise, the tag *unclear* was assigned to the item and it was removed from future analysis.

#### Annotation scheme.

Participants’ responses were assigned one of five tags.

The category *correct_reasoning* was assigned to hypothetical reasoning about alternatives of the kind described by the RSA model. An example for the top panel of Figure 1 would be “The speaker could have used the message *green monster* if they meant to refer to the other creature with the red hat, but they didn’t, which makes me think that they wanted me to choose the robot”.

Random responses were assigned to the category *guess*. Some participants indicated screen location as an explanation (often choosing the middle option) or referred to their responses on previous trials (e.g., “I have chosen a purple monster a lot in the past so now I will choose the robot”). Those were categorized as guessing since participants indicate that they had no way of differentiating between the target and the competitor and then used some other superficial criterion to break the tie.

Cases where participants described a reason for their choice which was something other than hypothetical reasoning about alternatives were labeled *other_reason*. Within that category, we further assigned one of the following five subcategories tothe explanations. The tag *visual_resemblance* was assigned when the participant stated that they selected the creature which they found to be “visually the most similar” to the message. Some of the explanations included a justification revealing the nature of the similarity the participant picked up on. For instance, a common reason for selecting the target in the top panel of Figure 1 was that the robot’s hat is facing in the same direction as the message, whereas the competitor’s hat is facing the other way. *odd_one_out* was assigned when the participant reported selecting a creature that stands out because it is the only one that has a certain feature. For instance, a participant might select the distractor in the top panel of Figure 1 as it is the only creature with a scarf and without a hat. The tag *salience* was assigned when the participant reported selecting a certain creature because it stood out to them. An example response for the top panel of Figure 1 which was assigned the *salience* tag is selecting the competitor “Because it is the biggest picture with a red cap”. The subcategory *salience* is quite closely related to *odd_one_out* since both of those correspond to selecting a response because it stands out. The difference is that in the *odd_one_out* case, a selection is made based on a creature being different from the other two based on a feature (exact match or mismatch), as opposed to being bigger or brighter, which is a relative difference. Often *odd_one_out* corresponded to selecting the distractor and adapting an inverse interpretation of the speaker’s message, e.g., using the red hat message to refer to the only creature that *is not* wearing a red hat. The tag *preference* was assigned when the explanation constituted a personal preference like “Robots are cool”.

One could argue that *preference* should be categorized as guessing since the decision did not involve reasoning about why the speaker sent a given message but instead broke the tie using the participant’s own preference. Our motivation was the following: guessing is by its nature not consistent, so we assigned an explanation to the *guess* category if, when presented with the same trial again, possibly with different randomization, the participant could have made a different selection. In the case of selecting a creature because it’s in the middle, since the screen location is randomized, a different creature could have been in the middle. In the case of a personal preference for robots, however, we assume that the participant would have consistently selected the robot in this situation if presented with this trial again.

Responses that involved a strategy that did not fall into one of the aforementioned categories were assigned to the subcategory *other*. An example would be a participant attempting to reason but their reasoning not being sound. For the bottom panel of Figure 1, incorrect reasoning could be selecting the competitor since “if [the speaker] had meant to communicate one of the other creatures, they would have used the purple monster message”.

Sometimes participants’ answers revealed that they misunderstood the instructions and took the fact that a feature is inexpressible (i.e., that it cannot be referred to directly) to mean that a creature that has the inexpressible feature could not be referred to at all. An example of an answer in that category for the top panel of Figure 1 would be “The speaker could not choose the robot so it must be the green monster”. Such responses were labelled *misunderstood_instructions*.

Answers where it was unclear what the participant meant, e.g., very brief answers just stating their selection (“The robot”) were labeled *unclear* and excluded from further analysis. We also excluded items where in their explanation the participant changed their mind as to their answer since that indicates that their explanation does not reflect their original reasoning strategy.

We investigated how internally consistent participants were when they saw one of the experimental items again during the strategy elicitation part of the experiment. We’d expect people who used *correct_reasoning* to be consistent, while *guess*ers may have picked the target the first time and the competitor the second time, or the other way around. That is indeed what we find. In the simple condition, collapsing across experiments since the pattern is very similar for all experiments, 78 (88.6%) participants who used the *correct_reasoning* strategy are consistent, selecting the target both times, and the remaining 10 (11.4%) selected the competitor the first time and the target the second time around. As predicted, *guess*ers are more mixed: 17 (32%) participants selected the target both times (presumably by chance), 15 (28.3%) participants selected the competitor both times, and the remaining 21 (39.6%) were inconsistent. Those breakdowns of responses were significantly different, with *correct_reason*ers consistently selecting the target both times more often (*χ*^2^(2, *N* = 141) = 52.63, *p* < 0.0001).

Among *other_reason* responses, it is notable that 33 (86.8%) participants who relied on *visual_resemblance* picked the target, 3 (7.9%) picked the competitor both times, and 2 (5.3%) were inconsistent. Other *other_reason* subcategories were pretty mixed.

In the complex condition, 44 (84.6%) *correct_reason*ers picked the target both times and 8 (15.4%) were inconsistent and picked the target the second time. *guess*ers, on the other hand, are pretty evenly split, with 34 participants (37.8%) selecting the target both times, 29 (32.2%) of participants selecting the competitor both times, and 27 (30%) inconsistent participants. The difference between *correct_reason*ers and *guess*ers is again significant (*χ*^2^(2, *N* = 142) = 32.77, *p* < 0.0001).

It is worth asking how reliable strategy explanations obtained through introspection are. Post-hoc explanations have been criticized for not always accurately reflecting the reasoning in the moment (Cushman, 2020). Nisbett and Wilson (1977) argued that humans lack access to introspective processes in the moment and therefore post-hoc explanations are rationalizations made after the fact. As an example, in the Wason selection task, people appear to be influenced by what has been termed textitmatching bias: when they are asked to indicate which cards need to be turned over to verify a logical rule (e.g., “If a card has a D on one side, it has a 3 on the other”), they are more like to select a card if it was mentioned before (a D or a 3 in this example). However, subjects appear to not be conscious of this bias, as it never comes up in post-hoc explanations (Evans, 2019).

We argue that, despite these limitations, post-hoc explanations have the potential to yield important insights. In our experiments below, we show that strategy elicitation can help reveal certain biases in the task, which some subjects are conscious of and which may be influencing other subjects subconsciously. We also see that people whose explanation featured *correct_reasoning* tend to be quite consistent in that they select the target both times when they see the same trial twice, and in fact a lot more consistent than those who reported guessing. Later in the paper we also show that there is considerable alignment between reported strategies and performance once biases present in the stimuli are accounted for. So whether or not the *correct_reasoning* explanation reflects correct hypothetical reasoning about alternatives about in the moment, it does seem to be a good predictor of solving the task correctly. Also, in these experiments we combine analysis of strategy explanations with careful manipulation of the stimuli and comparison of effect sizes. The fact that these two measures together yield consistent results gives more confidence in the validity of the provided explanations.

## EXPERIMENT 1: REPLICATION OF FRANKE AND DEGEN (2016) WITH REASONING ELICITATION

In this experiment, we replicated the reference game experiment by Franke and Degen (2016), additionally eliciting participants’ reasoning strategies in order to get an insight into how participants solve the task.

### Participants

60 native speakers of English with an approval rating of at least 95%, were recruited through the crowdsourcing platform Prolific.

### Methods

Participants completed the reference game described in Reference game. After completing the main experiment, participants saw one simple and one complex item again, presented in randomized order. When they made their selection, a red box appeared around it, along with the question “Why did you make that choice?” and a textbox. Participants’ strategies were then annotated as described in Annotation scheme.

### Results

One participant’s data was not saved on the server. Two further participants were excluded from analysis for not having paid enough attention because their performance on the unambiguous filled trials was below 80%. The data from the remaining 57 participants entered the analysis.

Franke and Degen (2016) fit a logistic mixed-effect regression model to verify that participants perform significantly better on the simple condition than they do on the complex, and that their performance on the complex condition is above the chance baseline (the ambiguous filler condition). The model in Franke and Degen (2016) included the maximal effect structure that allowed it to converge, which, in addition to per-participant random intercepts, included random slopes for message type (accessory or species) and trial number. It did not include a random slope for condition, or a per-item random intercept, presumably for convergence reasons. We believe, however, that it is important to include a random slope for condition in the model since the motivation for individual-level modeling is that individual participants may perform differently in the two conditions.

We also faced convergence issues when attempting to fit generalized linear mixed-effects models for our data despite using an optimizer (*bobyqa*, Powell (2009)): the only random effect structure with which the models for all 4 of our experiments converged was one that included only per-participant random intercepts. Therefore, in order to be able to keep the random effect structure maximal, we fit all our models using Bayesian regression using the *brms* package in R (Bürkner, 2017). Like in Franke and Degen (2016), we exclude from analysis trials on which distractors were selected (for Experiment 1, that corresponds to 2.1% of trials) and regress the binary correctness variable (whether target or competitor was selected) onto condition (simple, complex, or ambiguous, dummy-coded, with complex as the reference level)^1^, trial number, the interaction between trial number and condition, message type (creature or accessory), and position of the target creature on the screen (left, center, or right, dummy-coded, with left as the reference level). The random effect structure was maximal and included per-participant random intercepts and random slopes for condition, message type and trial number, and per-item random intercepts. The results are reported in Table 1.

**Table 1.** Effect size estimates, standard errors, and 95% confidence intervals for the 4 experiments, as well as effect size estimates, standard errors, and *p*-values for the original study by Franke and Degen (2016). Participants who did not reach 80%accuracy threshold on unambiguous trials were excluded from analysis.

	**F & D (2016) (51)**	**replication (57)**	**remapped (55)**	**all messages (56)**	**shapes (60)**
Intercept	−0.15 (0.11), *p* = 0.18	−0.29 (0.27), [−0.83, 0.24]	0.33 (0.17), [−0.00, 0.66]	−0.21 (0.21), [−0.63, 0.21]	0.20 (0.20), [−0.20, 0.61]
condition (simple vs. complex)	1.28 (0.12), *p* < 0.0001	2.16 (0.37), [1.47, 2.94]	0.46 (0.33), [−0.17, 1.15]	1.15 (0.26), [0.66, 1.68]	1.24 (0.30), [0.67, 1.86]
condition (ambig vs. complex)	−0.44 (0.13), *p* < 0.001	−0.19 (0.27), [−0.74, 0.34]	−0.33 (0.17), [−0.68, 0.01]	−0.06 (0.24), [−0.54, 0.41]	−0.42 (0.21), [−0.85, −0.03]
trial number	0.00 (0.00), *p* < 0.3	0.00 (0.01), [−0.01, 0.01]	−0.00 (0.00), [−0.01, 0.01]	−0.00 (0.00), [−0.01, 0.01]	0.01 (0.00), [0.00, 0.02]
simple vs. complex: trial	0.00 (0.01), *p* < 0.9	0.00 (0.01), [−0.01, 0.02]	0.02 (0.01), [0.01, 0.04]	−0.01 (0.01), [−0.02, 0.01]	−0.02 (0.01), [−0.03, −0.00]
ambig vs. complex: trial	0.01 (0.01), *p* < 0.33	0.00 (0.01), [−0.01, 0.02]	−0.01 (0.01), [−0.02, 0.01]	0.01 (0.01), [−0.01, 0.02]	−0.02 (0.01), [−0.03, −0.00]
target pos (middle vs. left)	1.28 (0.14), *p* < 0.0001	0.52 (0.15), [0.23, 0.80]	0.20 (0.14), [−0.07, 0.47]	0.30 (0.13), [0.04, 0.56]	0.38 (0.13), [0.11, 0.64]
target pos (right vs. left)	0.74 (0.13), *p* < 0.0001	0.54 (0.15), [0.25, 0.83]	0.09 (0.13), [−0.17, 0.34]	−0.20 (0.13), [−0.46, 0.06]	−0.04 (0.14), [−0.31, 0.23]
msg type (accessory vs. species)	−0.02 (0.12), *p* < 0.85	0.25 (0.21), [−0.15, 0.68]	−0.19 (0.14), [−0.48, 0.09]	0.48 (0.20), [0.08, 0.89]	0.22 (0.13), [−0.03, 0.47]

As in the original study by Franke and Degen (2016), in the replication we find strong evidence that participants performed better on the simple trials than on the complex ones (*β* = 2.16 (0.37), 95% CI [1.47, 2.94]); the effect size estimate is larger than in the original study (2.16 vs. 1.28). We do not find a significant effect of the difference between the population-level performance on the complex trials and the chance baseline (*β* = −0.19 (0.27), 95% CI [−0.74, 0.34], whereas in the original study, that difference was significant (*β* = −0.44 (0.13), *p* < 0.001). We think that this difference may in part be due to a different participant sample, but also due to the original study using a different strategy to code the ambiguous condition. Since in the ambiguous condition, two of the three creatures are identical, it needs to be decided somehow which one is target and which one is competitor. Since Franke and Degen (2016) report an uneven split in the ambiguous condition (46% of target choices vs. 51% of competitor choices), it appears that their implementation was to randomly decide on each trial which one of the two identical creatures was target and which one was competitor (let’s call this method coin-flipping). We, on the other hand, ensure an even split at the population level by having exactly half of the ambiguous non-distractor responses be correct. We chose this randomization method since we believe that it is a better approximation of a chance baseline and has smaller variance depending on the initialization^2^. Our simulation results suggest that the difference between the ambiguous and the complex conditions in the original study was amplified by the fact that Franke and Degen (2016)’s random initialization happened to result in more competitor responses.

Like in the original study, we find evidence of participants being more likely to select the target if it was in the center (*β* = 0.52 (0.15), 95% CI [0.23, 0.80]]) or on the right (*β* = 0.54 (0.15), 95% CI [0.25, 0.83]). We find no evidence of aneffect of trial or of the interaction between trial and condition, indicating absence of learning effects, nor an effect of message type (creature or accessory).

We now take a look at the annotations. Inter-annotator agreement was substantial, Cohen’s *κ* = 0.65, 95% CI [0.56, 0.75]. In the simple condition, 1 response labeled *exclude* and 8 responses labeled *unclear* out of 57 annotations were excluded. 48of the 57 annotations (84%) entered the analysis. In the complex condition, 2 *exclude* and 5 *unclear* responses were excluded, and 50 of the 57 annotations (88%) entered the analysis.

As can be seen in the top panel of Figure 3, 27.5% of correct responses (and 27.1% of total responses) in the simple condition fell into the *other_reason* category, suggesting that some participants arrived at the correct answer not via the assumed reasoning. When we examine the correct *other_reason* responses (bottom panel of Figure 3), we see that the majority of them used the *visual_resemblance* strategy. For the remainder of this paper, we will be discussing the simple condition, which turned out to be more susceptible to bias and more helpful for revealing it, but corresponding graphs for the complex condition can be found in the Appendix.

**Figure 2.**
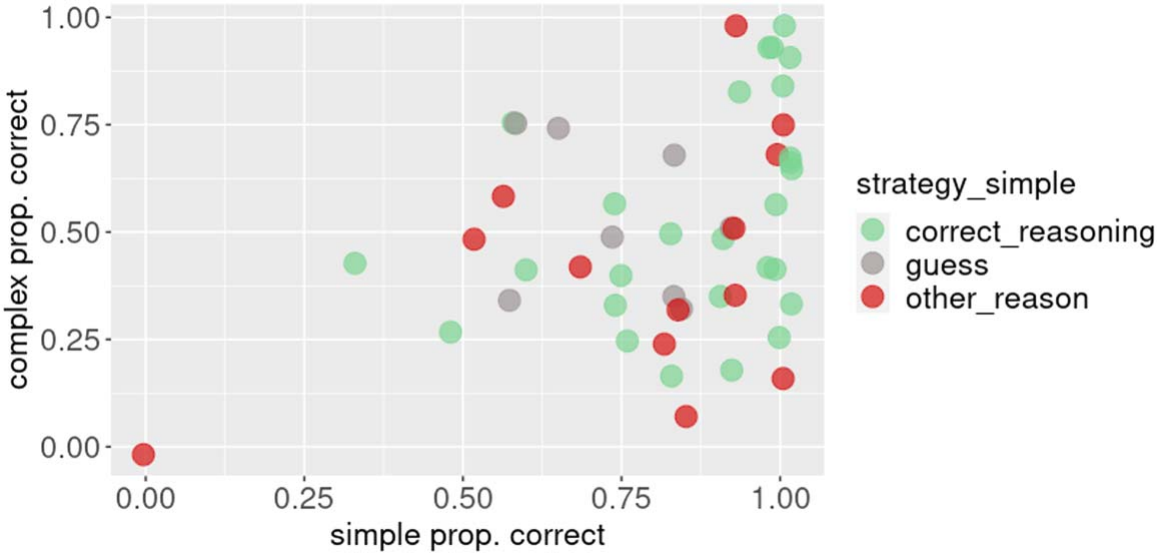
**Each participant’s average performance in Experiment 1, with the color corresponding to the strategy label on the simple condition.** The red dots correspond to participants who applied a strategy other than *guess* or *correct_reasoning*. Quite a few of the red dots are pretty far on the right, indicating that these participants got a trial correct for the wrong reason at least some of the time; in other words, these participants’ performance is likely inflated. Based on the performance alone, however, they are indistinguishable from correct reasoners.

In Figure 2, we plot participants’ average performance on the simple and complex trials. We see that the majority of participants who gave an *other_reason* explanation in the simple condition exhibit near-ceiling performance, so based on performance alone they would be indistinguishable from correct reasoners, although their answers are correct due to factors unrelated to RSA-style reasoning about alternatives at least some of the time.

**Figure 3.**
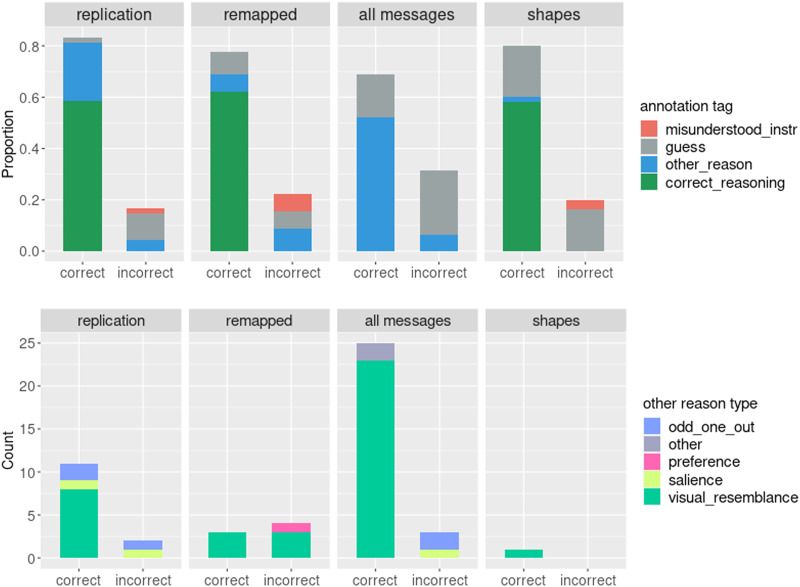
Participants’ explanations by tag in the simple condition (top – all explanations, bottom – *other_reason* explanations).

## EXPERIMENT 2: REMAPPED VERSION

We observed that in the original stimuli, the pairs of expressible features (that is, the features for which there is a message available to the speaker) constitute a kind of conceptual grouping – the two monsters are expressible as messages but the robot is not, the two hats are expressible as messages but the scarf is not.

The inexpressible features, therefore, because they are the odd-ones-out – robot is the only non-monster creature and the scarf is the only non-hat accessory – may stand out a lot more. To further probe the effect of stimuli on this task, we, therefore, swapped the expressible and inexpressible features around, breaking this conceptual grouping: the robot and the scarf were swapped for the purple monster and the blue hat respectively. The swapping is illustrated in Figure 5: along the creature dimension, we made the robot expressible and the purple monster inexpressible instead, and along the accessory dimension, we made the scarf expressible and the blue hat inexpressible instead.

**Figure 4.**
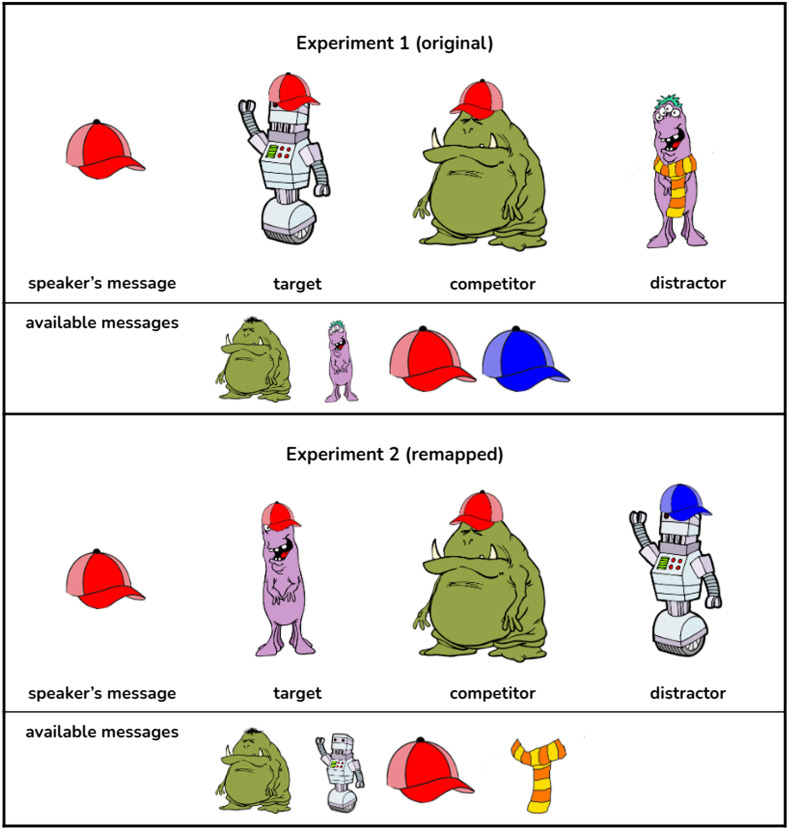
**An example of a remapped trial (the simple condition from Figure 1).** Because the robot gets swapped with the purple monster, the target becomes purple monster with the red hat, the competitor stays the same, and the distractor becomes a robot(swapped with the purple monster) with a blue hat (swapped with a scarf).

So in this experiment, the simple trial in Figure 1 looks as follows (illustrated in Figure 4): the message stays the red hat since no swapping had been performed there; the target is now a purple monster with a red hat (since we swapped the robot and the purple monster around), the competitor remains the same since no swapping has been performed for either the green monster or the red hat, and the distractor is now a robot with a blue hat (since the purple monster is swapped with the robot and the scarf is swapped with the blue hat). Therefore, underlyingly, all trials remained the same as in Experiment 1, the only difference is that the inexpressible features are represented with different images. We used the images from the original study so no changes were performed e.g., to the hat orientation.

**Figure 5.**
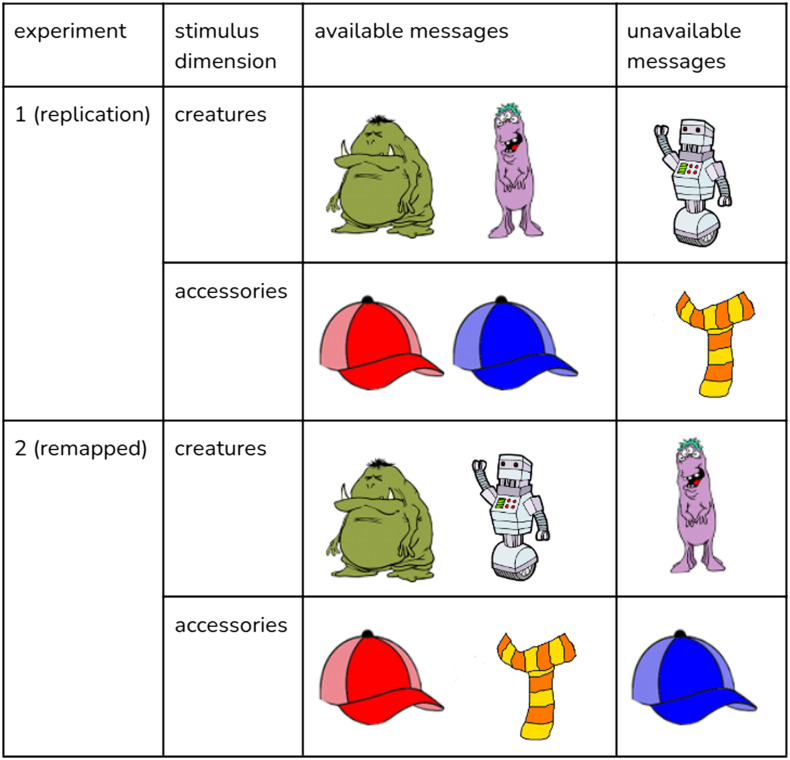
For Experiment 2, we changed which features were expressible as messages.

### Participants

60 native speakers of English with an approval rating of at least 95%, were recruited through the crowdsourcing platform Prolific.

### Methods

We changed which features were expressible as messages, swapping the robot and the scarf for the purple monster and the blue hat respectively. Otherwise the experiment was identical to the replication. An example of how the swapping was performed is shown in Figure 4.

### Results

One participant’s data was not saved on the server. Four further participants were excluded from analysis for not having paid enough attention because their performance on the unambiguous filler trials was below 80%. The data from the remaining 55 participants entered the analysis.

As can be seen in Table 1, we observe a stark change in the effect for simple vs. complex condition: the effect size is a lot smaller than in the replication, and the effect is no longer significant (*β* = 0.46 (0.33), 95%CI [−0.17, 1.15]). Like in the replication, performance on the complex condition is not significantly different from the chance baseline (*β* = −0.33 (0.17), 95% CI [−0.68, 0.01]).

Strategy responses were again annotated by two annotators, one of whom was blind to the purpose of the experiment. Inter-annotator agreement was again substantial, Cohen’s *κ* = 0.77, 95% CI [0.68, 0.86]. In the simple condition, 4 responses labeled *exclude* and 6 responses labeled *unclear* out of 55 annotations were excluded. 45 of the 55 annotations (82%) entered the analysis. In the complex condition, 3 *exclude* and 10 *unclear* responses were excluded, and 42 of the 55 annotations (76%) entered the analysis.

When we examine the breakdown of annotation tags (Figure 3), we see that a smaller proportion of correct answers and a larger proportion of incorrect answers is comprised of *other_reason* responses, and while in the original study, *visual_resemblance* led participants to always select the correct answer, in the remapped version, it was now incorrect some of the time; salience, preference, and *odd_one_out* reasoning now pointed participants to the competitor instead of the target. For instance, in the simple trial depicted in Figure 4, in the original version, the creature that is the odd-one-out and therefore a more salient one is the robot, which happens to be the target, so if a participant selects it based on *salience* or *odd-one-out* reasoning, they will happen to be correct. In the remapped version, on the other hand, the target is less visually salient because it is one of the two monsters, therefore, the same *salience* or xtitodd_one_out reasoning may lead the participant to select the distractor, the robot, which would be incorrect. This suggests that in the remapped version of the task, some of the existing biases in the stimuli were now having the opposite effect from the original study, that is, “deflating” participant performance.

## EXPERIMENT 3: ORIGINAL EXPERIMENT, ALL MESSAGES AVAILABLE

We wanted to know how often participants would make the right choice coincidentally in the original experiment, in a setting where correct hypothetical reasoning about alternatives is made impossible, thus isolating stimuli effects.

In order to isolate stimuli effects and make *correct_reasoning* impossible, we made all six features expressible. As a result, *correct_reasoning* is no longer possible in the simple condition, since there are now unambiguous messages available to refer to the target as well as the competitor. For example, in the top panel of Figure 1, both the target and the competitor can now be referred to unambiguously via the messages “robot” and “green monster” respectively, so the message “red hat” is now completely ambiguous.

Note that in the complex condition, *correct_reasoning* is still possible because the target still cannot be unambiguously identified by a message, while both the competitor and the distractor can be.

Thus, in this case, none of the formal models should be able to solve the simple implicature condition and, like in the original setup, only *L*_2_ should be able to solve the complex one.

### Participants

60 native speakers of English with an approval rating of at least 95%, were recruited through the crowdsourcing platform Prolific.

### Methods

One participant’s data was not saved on the server. Three further participants were excluded from analysis for not having paid enough attention because their performance on the unambiguous filled trials was below 80%. The data from the remaining 56 participants entered the analysis.

Apart from the feature expressibility manipulation, the experiment was identical to the replication.

### Results

Table 1 shows that, with all messages available, the correct option is still selected for reasons that are not correct hypothetical reasoning quite often, as evidenced by the larger significant effect of condition (complex vs. simple, *β* = 1.15 (0.26), 95% CI [0.66, 1.68]), notably, considerably more often than in the remapped experiment (*β* = 0.46 (0.33), 95% CI [−0.17, 1.15]).

Inter-annotator agreement was again substantial, Cohen’s *κ* = 0.67, 95% CI [0.57, 0.78]. In the simple condition, 1 response labeled *exclude* and 7 responses labeled *unclear* out of 56 annotations were excluded. 48 of the 56 annotations (86%) entered the analysis. In the complex condition, 6 *unclear* responses were excluded, and 50 of the 56 annotations (89%) entered the analysis.

When we take a look at the performance by tag (Top panel of Figure 3), we see that when participants used an *other_reason* strategy in the simple condition, the vast majority of the time (89.2% of cases) their strategy coincidentally led them to select the target. When we examine the *other_reason* tags in more detail, we see that the strategy that resulted in accidental correct responses is *visual_resemblance*, that is, similarity of the target to the message based on the head of a scarf-wearing creature being uncovered, similar to the message (that creature without an accessory) or the orientation of the hat message matching that of the referent.

This corroborates the claim that factors unrelated to reasoning, most importantly incidental visual resemblance of the message to the target, inflate performance in the original experiment.

## EXPERIMENT 4: MITIGATING BIAS BY USING ABSTRACT STIMULI

Having shown that the visual resemblance bias accounts for the vast majority of coincidental target choices, we repeated the original experiment with a version of the stimuli that we hoped would be less susceptible to the *visual_resemblance* bias.

### Participants

60 native speakers of English with an approval rating of at least 95%, were recruited through the crowdsourcing platform Prolific.

### Methods

There were no exclusions based on accuracy in this experiment, as all 60 participants scored above 80% on the unambiguous filler trials.

Having identified the biases in the original stimuli, we attempted to mitigate them. We hypothesized that using more abstract stimuli, of the kind used in the original RSA study (Frank & Goodman, 2012), would not create opportunity for a visual resemblance bias between the message and the target.

We therefore ran a version of the experiment with abstract stimuli, using geometric shapes and colors instead of creatures and accessories: square, triangle and circle corresponding to the robot, green monster and purple monster respectively, and the colors blue, green and red corresponding to the scarf, red hat and blue hat respectively. When the message was a color, it was represented by a color tube, and when it was a shape, it was represented by a shape contour. An example of trial from Experiment 4, corresponding directly to Figure 1 from Experiment 1, is included in Figure 6.

**Figure 6.**
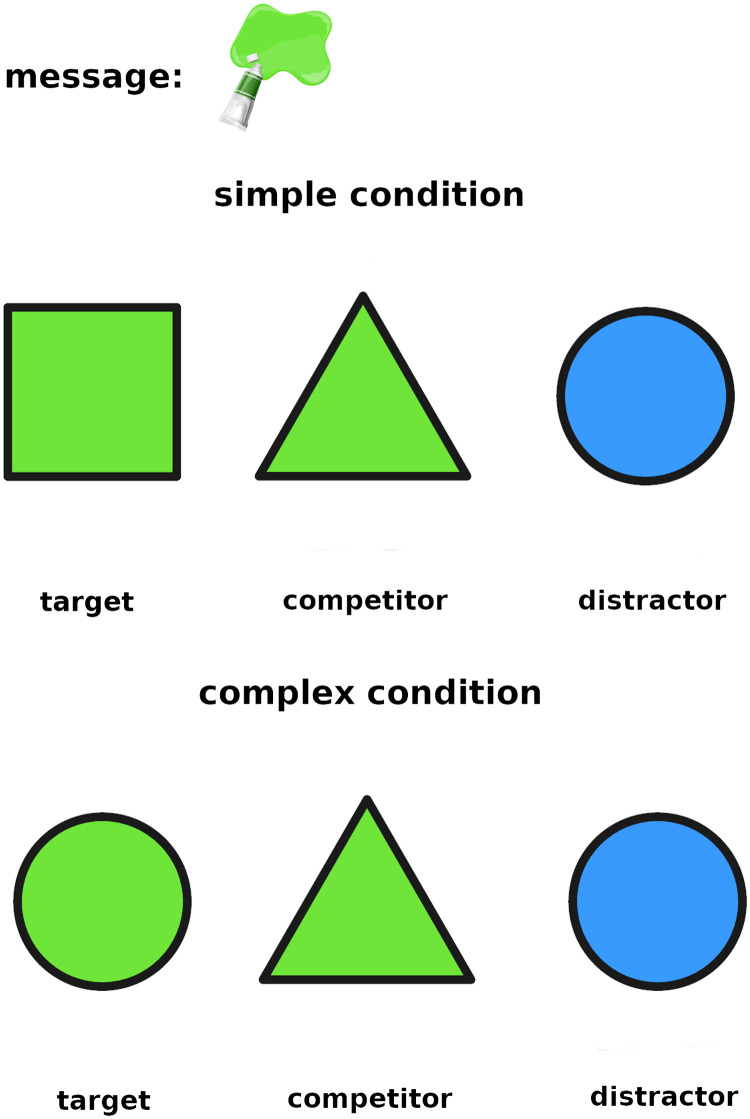
**Example of a simple and a complex trial for Experiment 4.** These trials directly correspond to those depicted in Figure 1 for Experiment 1.

Thus, underlyingly, the experiment remained the same as the original, the only difference being what images were used to represent the messages and the referents.

### Results

Inter-annotator agreement was again substantial, Cohen’s *κ* = 0.80, 95% CI [0.71,0.89]. In the simple condition, 2 responses labeled *exclude* and 3 responses labeled *unclear* out of 60 annotations were excluded. 55 of the 60 annotations (92%) entered the analysis. In the complex condition, 1 *exclude* and 6 *unclear* responses were excluded, and 53 of the 60 annotations (88%) entered the analysis.

We see in Figure 3 that with abstract stimuli, barely any *other_reason* explanations are given, indicating that people do not seem to rely on clues like visual similarity but instead employ either guessing or correct hypothetical reasoning about alternatives, as assumed by formal probabilistic models. Only one *visual_resemblance* response is given, and it does happen to accidentally lead to selecting the target: when the message is a circle, the blue circle isselected with the justification that the blue color is “nearest to clear”, so a clear circle is most similar to the blue circle.

We conclude the abstract stimuli appear to be less susceptible to biases that inflated performance on the original experiment, and in particular, the visual resemblance bias. Therefore, we can have more confidence that the task results more accurately reflect participants’ pragmatic reasoning ability. In terms of the effect sizes, this study’s results closely resemble those of the original Franke and Degen (2016) study and our replication: *β* = 1.24 (0.30), 95% CI [0.57, 1.86] for simple vs. complex condition in this study vs. *β* = 2.16 in the replication; like in the original study and unlike in our replication, there is evidence for a difference between the complex condition and chance (*β* = −0.42 (0.21), 95% CI [−0.85, −0.03]). Thus, it appears that the original study, despite the biases, fairly accurately captures the effects at the population level. However, now we can be more certain that they accurately reflect individual-level performance as well.

## DISCUSSION

In this study, we elicited participants’ strategies and manipulated which messages were expressible to probe the stimuli first introduced by Degen and Franke (2012) and used in Degen et al. (2013) and Franke and Degen (2016) for biases. Effect sizes varied greatly between experiments and appear to be inflated in the original task by factors unrelated to reasoning. The most prominent bias was visual resemblance, where participants selected the creature most similar to the message, which in most cases happened to be the target. We then replicated the experiment with a version of the stimuli that we showed to be less susceptible to the visual resemblance bias.

There remains the question of whether the effect sizes in Experiments 1 and 4 are even comparable: the stimuli in the original task have biases, inflating the effect of condition, but they are also visually more complex, potentially leading to higher task difficulty and lower effect of condition than the abstract stimuli in Experiment 4. In other words, there appears to be no way to know exactly how much smaller the “true” effect size of condition in the original experiment would have been if there were no stimuli-related biases, because when we use different stimuli that do not have the biases, those stimuli also have different perceptual properties which affect task complexity, and, correspondingly, effect sizes. We would expect the “true" effect sizes to lie somewhere between those in Experiment 1 and Experiment 2, since in the original experiment, biases inflate participants’ performance, and in the remapped version (Experiment 2), biases still exist but they lead participants to choose incorrectly.

The final experiment, which used different stimuli, was shown to be much less susceptible to biases present in the original stimuli. That means that we can have more confidence that participants’ performance on the task is a more reliable reflection of their reasoning ability, at the population level, and especially at the individual level.

To investigate individual-level performance in Experiments 1 and 4 in more detail, we ran Latent Profile Analysis on participants’ average performance on simple and complex conditions using the *tidyLPA* package in R to obtain reasoner classes. We decided to use LPA and not the Bayesian model from Franke and Degen (2016) because the latter is too strict for our purposes: it presupposes the existence of only three classes (the theoretically motivated classes *L*_0_, *L*_1_, and *L*_2_), and since we saw that many participants used other strategies, we wanted to have a data-driven way of identifying classes which may potentially include classes other than the ones corresponding to the three probabilistic listener models.^3^ Recall that Experiments 1 and 4 are underlyingly the same and the only difference is what images are used as the representation of the messages and referents, hence *L*_0_, *L*_1_ and *L*_2_ RSA models make the same predictions for each trial of the two experiments.^4^

**Figure 7.**
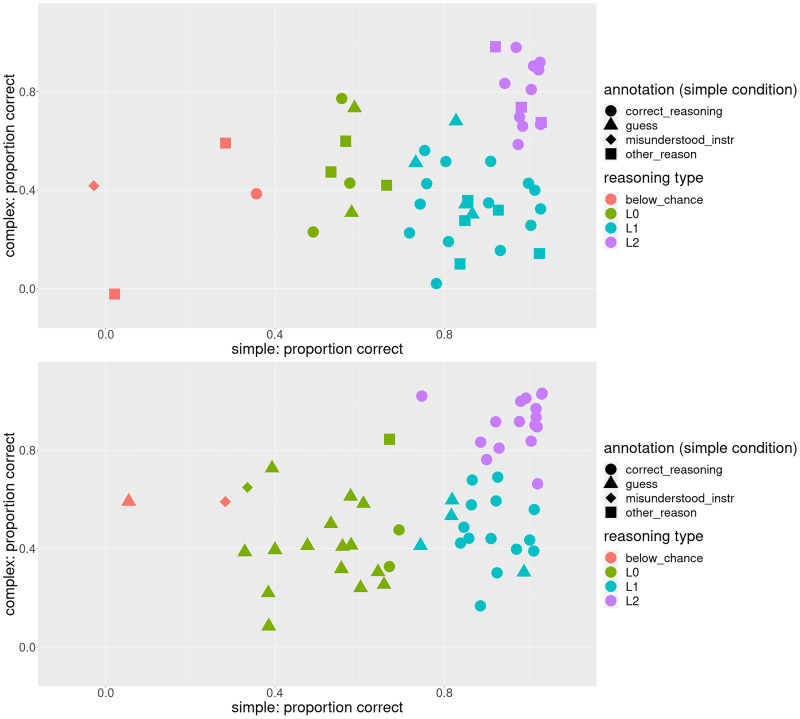
**Classes of participants identified by LPA (top – Experiment 1 (replication), bottom – Experiment 4 (debiased stimuli)).** Three of the identified classes approximately correspond to the theoretical predictions of listener models *L*_0_, *L*_1_, and *L*_2_. The fourth LPA class, which is the smallest for both experiments, corresponds to participants who perform below chance level in the simple condition.

For both experiments, the best fit, as measured by AIC and BIC, was obtained by models with 4 classes (model fit for each number of classes can be found in the Appendix).^5^ As can be seen in Figure 7, three of the four identified classes approximately correspond to the predictions of the three formally defined reasoning types from Franke and Degen (2016), *L*_0_ (at chance on both conditions), *L*_1_ (can solve the simple but not the complex trials), and *L*_2_ (can solve both conditions). Additionally, there’s the smallest fourth class, consisting of 4 and 2 participants respectively for the two experiments, of participants who perform below chance on the simple condition and at or below chance on the complex. Strategy annotations also approximately match our expectations, but the pattern is more pronounced for Experiment 4: participants assigned to the class *L*_0_ guess in the simple condition while participants assigned to classes corresponding to *L*_1_ and *L*_2_ apply correct reasoning in the simple condition. The corresponding scatterplot for the complex condition can be found in the Appendix.

We see that in Experiment 1, of the participants whose strategy in the simple condition was labeled *other_reason*, 3 are assigned to the *L*_0_ class, 5 to *L*_1_, and 3 to *L*_2_. As we saw in the above experiments, these participants’ performance was likely inflated by factors unrelated to reasoning; therefore, they were possibly estimated to have a more advanced reasoning type than they actually have. In Experiment 1, only one participant used an *other_reason* strategy in the simple condition; that participant was assigned to *L*_0_, although they are on the border with *L*_2_ (bottom panel of Figure 7). As we saw in Experiment 4, this participant happened to choose the target for the wrong reason (a creative application of the *visual_resemblance* strategy); therefore, their performance is also inflated. However, the fact that this is only 1 participant is a large improvement compared to Experiment 1.

**Figure 8.**
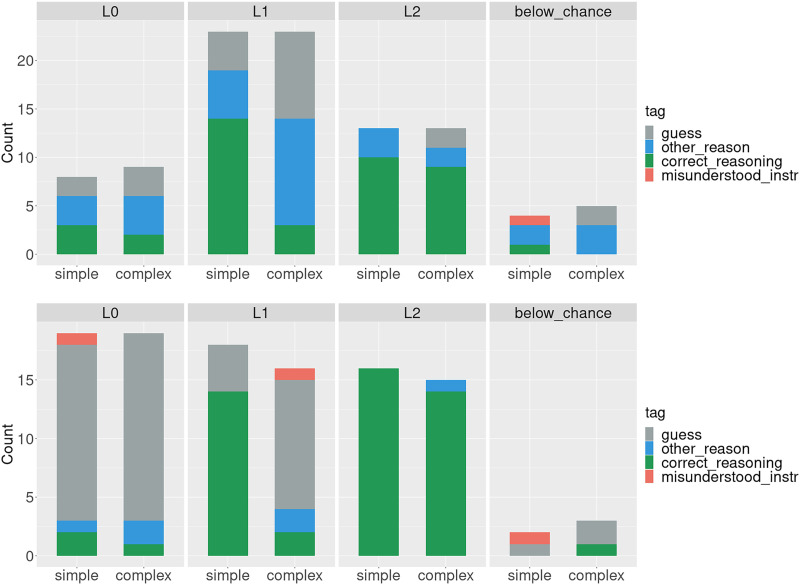
Tags in each condition for the classes identified by LPA (top – Experiment 1 (replication), bottom – Experiment 4 (debiased stimuli)).

When we look at the breakdown by tag for the identified classes (Figure 8), we see that the responses in Experiment 4 much more cleanly map onto the corresponding formal classes than the responses from Experiment 1 (*L*_0_ guesses forboth types of trials, *L*_1_ applies correct reasoning about alternatives for the simple condition and guesses on the complex, and *L*_2_ applies correct reasoning in both conditions), again supporting the claim that the debiasing of the stimuli was successful and constitute a better proxy for participants’ reasoning ability as defined by formal probabilistic models. In order to quantitatively corroborate this finding, we conducted cluster analysis. Each participant was assigned a class label based on performance. For that, we used the four classes identified by LPA: *L*_0_, *L*_1_, *L*_2_, and *other* (people who ended up in the fourth, *below_chance* class). Each participant was also assigned a class label based on the annotated strategies: *L*_0_ if they used the *guess* strategy in both conditions, *L*_1_ if their strategy was labeled *correct_reasoning* in the simple condition and *guess* in the complex, and *L*_2_ if their strategy in both condition was *correct_reasoning*. Finally, participants who used an *other_reason* strategy in either condition were given the label *other*. Participants whose strategy was labeled *unclear* or *exclude* were excluded from this analysis, resulting in 42 participants for Experiment 1 and 47 participants for Experiment 4 entering the analysis. The motivation for the *other* label is that we would ideally want people who use other reasoning to be distinguishable from the three reasoning types based on performance. In order to see how well the performance-based and annotation-based classes aligned, we computed cluster homogeneity and completeness for Experiment 1 and Experiment 4, with performance class labels as reference. For Experiment 1, homogeneity is 0.13 and completeness is 0.13, and for Experiment 4, homogeneity is 0.56 and completeness is 0.51. This further supports the observation that more abstract stimuli lead to a better alignment between performance and the underlying strategy.

While it is unrealistic to expect no stimuli effects, it is important to know what biases are present and how strong they are because that influences the conclusions that are drawn. This can be quite important, in particular, for individual difference studies, where spurious correlations of performance with individual measures may emerge or real ones may not be identified due to such stimulus effects. For instance, the study by Mayn and Demberg (2022), which explored individual differences that modulate participants’ reasoning complexity in the reasoning game and found an effect of non-verbal IQ and reflectivity, was conducted using the original biased stimuli, suggesting that it would be important to repeat the analysis and confirm that the findings still hold and that the effect of IQ and reflectivity is not driven or inflated by the stimuli biases.

We used post-hoc strategy elicitation to probe the experimental design of the reference game for biases. Post-hoc explanations have been criticized for not to always accurately reflecting the reasoning in the moment (e.g., Cushman, 2020). If we look at the relationship between participants’ performance and the elicited strategy explanation in Experiment 1 (Figure 2), we see that while most participants whose strategy explanation on the simple trial was labeled *correct_reasoning* performed near ceiling on average, there are a few participants who show chance-level average performance. One reason why this might be the case is that the fact that a participant applied a certain strategy, e.g., xtitcorrect_reasoning, on a given trial does not mean that they applied it successfully on all trials. Another possibility is that these participants were actually guessing in the moment and came up with the correct reason for their selection after the fact. This suggests that these explanations on a single trial are not a perfect proxy for participants’ reasoning strategies throughout the experiment. However, the fact that participants who provided a *correct_reasoning* reasoning on the simple trial have much better average performance than guessers (0.87 (0.17) vs. 0.66 (0.32)) suggests that, while imperfect, post-hoc explanations are a proxy for the strategies participants used during the experiment. While there are limitations to elicited post-hoc explanations, they can be a useful tool for revealing biases in an experimental design that could otherwise be missed, as was the case in this study.

Recently, Ryzhova et al. (2023) applied a method quite similar to the strategy elicitation method used in this work to a different pragmatic phenomenon, atypicality inferences, in order to gain more insight into how participants solved the task. When an event which is very predictable in a given context is overtly mentioned, it may be inferred that the event is actually atypical for the referent (e.g., “John went to the grocery store. He paid the cashier” may result in the atypicality inference that John does not usually pay at the grocery store). In addition to collecting ratings of how typical participants expected the redundantly mentioned activity to be for the referent, the authors asked participants to justify the ratings they gave. Based on the provided justifications, they were able to make a distinction between two groups of participants which would have been unidentifiable based on ratings alone: those who did not make the atypicality inference and those who initially made it but then rejected it (e.g., “Not paying would be stealing, therefore it’s unlikely that John does not pay”) and gave a high typicality rating. Adding explanations to the experimental measure allowed the authors to identify different reasoning which results in the same performance, much like in the current work where we show that high task performance may be the result of correct reasoning but also of unintended biases in the task where people get away with applying simpler strategies. The fact that strategy elicitation has proved useful for two different pragmatic phenomena – the reference game in our work and in the case of atypicality inferences – suggests that it can be useful more broadly and may be worth applying when investigating other pragmatic phenomena and seeking to better understand the processes underlying pragmatic reasoning.

More broadly, we believe that, when designing experiments, it is very important to ask the question how to make sure that the measure that is being used is indeed tapping into the construct of interest. Might there be other explanations for why participants are behaving a certain way? Here, we showed two ways that can be used for probing an experimental design: careful manipulation of the stimuli and strategy elicitation. Of course, what methods are appropriate depends on the experimental setup: for instance, strategy elicitation seems to mostly apply when the studied phenomenon is expected to involve relatively conscious reasoning or reflection; manipulating stimuli in systematic ways and examining how that affects the results seems to be applicable more broadly. There may be biases which are not reported because subjects are not aware of them, just like participants don’t report matching bias in Wason’s selection task. Therefore, we believe that it is worthwhile to explore the application of on-line measures, such as eye gaze, to getting a better sense of participant strategies, and learn more about how those relate to performance and explanations obtained through introspection. How people actually solve a task is a difficult question as we cannot look directly into people’s heads and have to rely on proxies like performance or explanations, but it is a very important one, and therefore we should make it a priority to search for possible ways to get closer to the answer.

## ACKNOWLEDGMENTS

We would like to thank the anonymous reviewers for their helpful comments and suggestions and AriaRay Brown for assistance with annotation and helpful conversations.

## FUNDING INFORMATION

This project is supported by funding from the European Research Council (ERC) under the European Union’s Horizon 2020 Research and Innovation Programme (Grant Agreement No. 948878).

## Notes

^1^ Franke and Degen (2016) use Helmert coding where simple is compared to the rest and complex is compared to ambiguous, but since *brms* uses dummy coding, we set complex to be the reference level so that we can obtain both of simple vs. complex and complex vs. ambiguous comparisons.^2^ When we ran each randomization method 100 times on our replication data, the standard deviations of effect size estimates were larger for the coin-flipping method. Related to that, the difference between the complex and the ambiguous conditions came out significant 10 out of the 100 times when the coin-flipping method was used and none of the times when we ensured a 50–50 split.^3^ Incidentally, applying Franke and Degen (2016)’s class assignment model to our data yielded uninterpretable classes.^4^ That is also true for Experiment 2, but not for Experiment 3, where neither *L*_1_ nor *L*_2_ are predicted to be able to solve the simple condition since it is made completely ambiguous.^5^ For Experiment 4, the 5-class model has the best fit according to AIC whilethe 4-class model has the best fit according to BIC. The only difference between those two clusterings is that for the 5-class model, one participant whose accuracy is 0 for both simple and complex is in their own separate class. Therefore, for comparability with Experiment 1, we use the 4-class model.

## Figures and Tables

**Figure 9.**
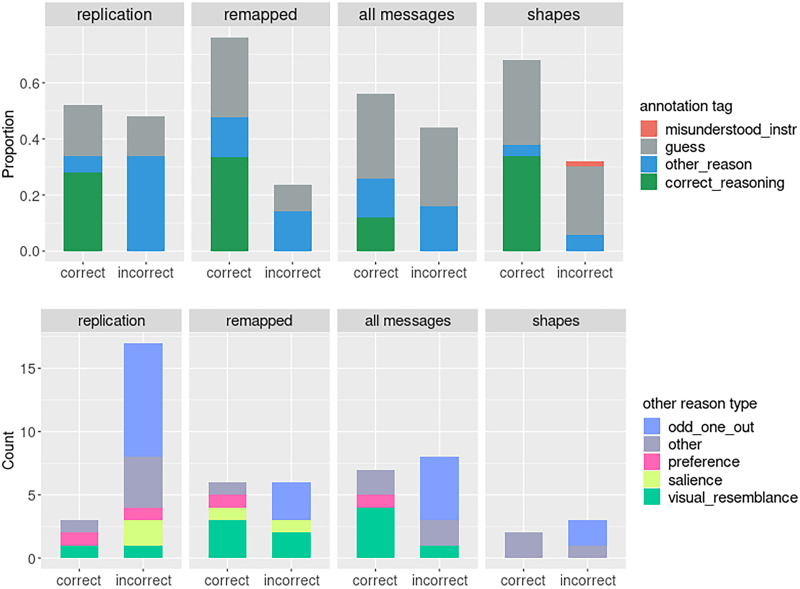
Participants’ explanations by tag in the complex condition (top – all explanations, bottom – *other_reason* explanations).

**Figure 10.**
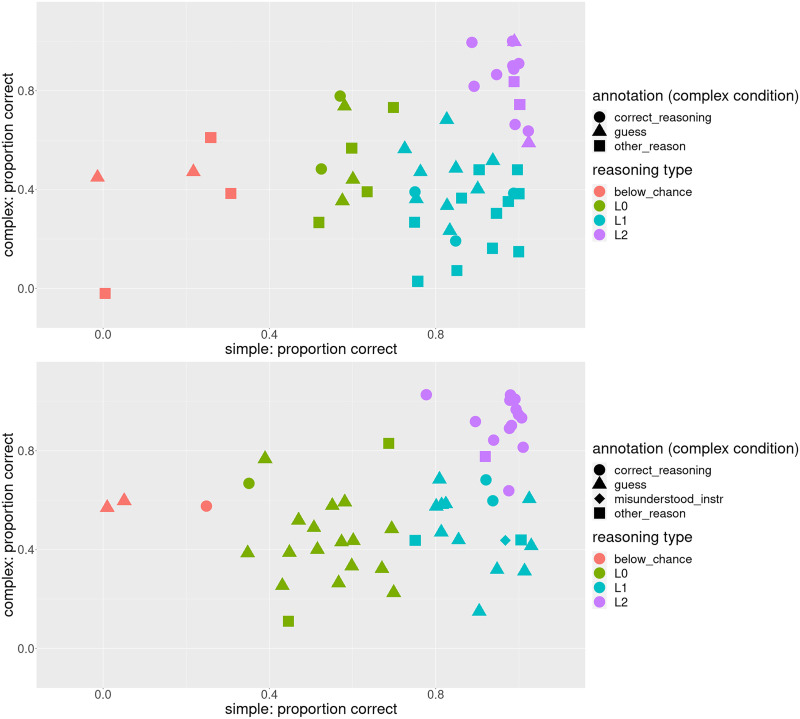
**Classes of participants identified by LPA (top – Experiment 1 (replication), bottom – Experiment 4 (debiased stimuli)).** Shapes correspond to the tags assigned to the reasoning strategies in the complex condition.

## APPENDIX

### Annotations for the Complex Condition

In Figure 9 we provide by-tag results in the complex condition for the two experiments. It is notable that there is a large proportion of *odd_one_out* responses. Those correspond to the participant interpreting the message inversely and selecting the distractor. For example, in the bottom panel of Figure 1, a participant might interpret the message “red hat” as meaning “the only creature without a red hat” and select the distractor, the purple monster with a scarf. The fact that we observe a smaller proportion of *other_reason* responses in Experiment 4 is a good sign because the employed strategies are predominantly *correct_reasoning* and *guess*, as is assumed by formal probabilistic models.

### LPA Model Fit

Table 2 and Table 3 show fits for models with 1 through 6 latent classes for Experiments 1 and 4 respectively, as measured by AIC and BIC. The 4-class model provides the best fit for Experiment 1, and the classes approximately correspond to the predictions of the three probabilistic listener models: *L*_0_, *L*_1_, and *L*_2_. For Experiment 4, the 4-class model has the best fit according to BIC and the 5-class model has the best fit according to AIC. The difference between the 4- and 5-class models is that in the 5-class model, one participant who utilized the *odd-one-out* strategy on all trials, resulting in 0 accuracy on both simple and complex trials, is assigned to their own class. Since that is the only difference, for comparability in the article we discuss 4-class models for both experiments.

**Table 2.** Fit of LPA models for Experiment 1.

**Num. classes**	**AIC**	**BIC**
1	13.89	22.06
2	−4.88	9.42
3	−6.37	14.06
4	**−18.83**	**7.73**
5	−16.55	16.14
6	−11.62	27.2

**Table 3.** Fit of LPA models for Experiment 4.

**Num. classes**	**AIC**	**BIC**
1	30.27	38.65
2	17.96	32.62
3	8.55	29.5
4	−6.16	**21.07**
5	**−9.66**	23.85
6	−4.87	34.92

### LPA Classes (Complex Condition)

The 4 latent classes for Experiments 1 and 4 with annotation tags from the complex condition are included in Figure 10. In Experiment 1 (top panel), we observe a large number of people who use the *odd_one_out* strategy, thus performing below chance. They are assigned to the class corresponding to the *L*_1_ model, where the reasoner can solve simple implicatures but guesses randomly on complex ones. That is not ideal since the *odd_one_out* strategy where the message is interpreted inversely is a different, more complex strategy than guessing. In Experiment 4 (bottom panel), we get cleaner results: there are only a few people who use *odd-one-out* reasoning; for the most part, *L*_0_ and *L*_1_ classes consist of people whose strategy in the complex condition was guessing, and the *L*_2_ class consists of people who applied the *correct_reasoning* strategy.
